# Remote Consulting in Primary Health Care in Low- and Middle-Income Countries: Feasibility Study of an Online Training Program to Support Care Delivery During the COVID-19 Pandemic

**DOI:** 10.2196/32964

**Published:** 2022-06-14

**Authors:** Andrew Downie, Titus Mashanya, Beatrice Chipwaza, Frances Griffiths, Bronwyn Harris, Albino Kalolo, Sylvester Ndegese, Jackie Sturt, Nicole De Valliere, Senga Pemba

**Affiliations:** 1 School of Public Health and Preventative Medicine Monash University Melbourne Australia; 2 Warwick Medical School University of Warwick Coventry United Kingdom; 3 Department of Public Health Faculty of Medicine St Francis University College of Health and Allied Sciences Ifakara United Republic of Tanzania; 4 Centre for Health Policy University of the Witwatersrand Johannesburg South Africa; 5 Florence Nightingale Faculty of Nursing, Midwifery and Palliative Care King's College London London United Kingdom; 6 Warwick Clinical Trials Unit University of Warwick Coventry United Kingdom

**Keywords:** remote consultation, mobile consulting, digital health, telehealth, mHealth, eHealth, mobile health, health care, cascade, train the trainer, low- and middle-income countries, rural areas, Tanzania, Kirkpatrick, consultation, training, low- and middle-income, rural, COVID-19

## Abstract

**Background:**

Despite acceleration of remote consulting throughout the COVID-19 pandemic, many health care professionals are practicing without training to offer teleconsultation to their patients. This is especially challenging in resource-poor countries, where the telephone has not previously been widely used for health care.

**Objective:**

As the COVID-19 pandemic dawned, we designed a modular online training program for REmote Consulting in primary Health care (REaCH). To optimize upscaling of knowledge and skills, we employed a train-the-trainer approach, training health workers (tier 1) to cascade the training to others (tier 2) in their locality. We aimed to determine whether REaCH training was acceptable and feasible to health workers in rural Tanzania to support their health care delivery during the pandemic.

**Methods:**

We developed and pretested the REaCH training program in July 2020 and created 8 key modules. The program was then taught remotely via Moodle and WhatsApp (Meta Platforms) to 12 tier 1 trainees and cascaded to 63 tier 2 trainees working in Tanzania’s rural Ulanga District (August-September 2020). We evaluated the program using a survey (informed by Kirkpatrick's model of evaluation) to capture trainee satisfaction with REaCH, the knowledge gained, and perceived behavior change; qualitative interviews to explore training experiences and views of remote consulting; and documentary analysis of emails, WhatsApp texts, and training reports generated through the program. Quantitative data were analyzed using descriptive statistics. Qualitative data were analyzed thematically. Findings were triangulated and integrated during interpretation.

**Results:**

Of the 12 tier 1 trainees enrolled in the program, all completed the training; however, 2 (17%) encountered internet difficulties and failed to complete the evaluation. In addition, 1 (8%) opted out of the cascading process. Of the 63 tier 2 trainees, 61 (97%) completed the cascaded training. Of the 10 (83%) tier 1 trainees who completed the survey, 9 (90%) would recommend the program to others, reported receiving relevant skills and applying their learning to their daily work, demonstrating satisfaction, learning, and perceived behavior change. In qualitative interviews, tier 1 and 2 trainees identified several barriers to implementation of remote consulting, including lacking digital infrastructure, few resources, inflexible billing and record-keeping systems, and limited community awareness. The costs of data or airtime emerged as the greatest immediate barrier to supporting both the upscaling of REaCH training and subsequently the delivery of safe and trustworthy remote health care.

**Conclusions:**

The REaCH training program is feasible, acceptable, and effective in changing trainees’ behavior. However, government and organizational support is required to facilitate the expansion of the program and remote consulting in Tanzania and other low-resource settings.

## Introduction

### Background

Essential health services are not available for over a third of the world’s population, and most of this population is in low- and middle-income countries (LMICs) [1]. Marginalized communities, including those living in rural areas and informal settlements or slums, have least access to high-quality health care [2]. High-quality care includes appropriate and timely treatment and follow-up [2], and its provision forms part of the United Nations Sustainable Development Goal for health [3].

Even prior to the COVID-19 pandemic, remote consulting was considered to have the potential to increase access to quality health care, especially in rural communities [4-6]. It is estimated that 85% of individuals across LMICs own a mobile phone [7]. Although Tanzania has lower rates of ownership, it still has 75% mobile phone ownership across the population and 90% among health workers [8]. Mobile phone ownership is lower amongst rural, older, illiterate, and female populations compared to other population groups but is rapidly increasing [4,7]. Patients find remote consulting acceptable and appreciate the consistency and continuity of care achieved through improved communication [9].

From the beginning of the COVID-19 pandemic, the World Health Organization recommended remote consultation using phones or videoconferencing as an option for protecting the safety of patients and health workers and to enable continued health care provision [10,11]. Worldwide, in the face of the pandemic, remote consulting increased but often with little preparation and training [12]. This lack of training in the use of health technology is a key barrier to the acceptance and uptake of remote consulting in LMICs [4,13], along with health workers’ worries about increasing personal workload [9].

Worldwide, continuing medical education delivered remotely has been shown to be acceptable, feasible, and desirable [14]. It enables greater geographic accessibility and time flexibility [15] and has been shown to be as effective as traditional teaching methods and far more effective than no training [16,17]. Issues of network connectivity, costs of data/airtime, access to electricity, and usability of the device are challenges that need to be addressed [18].

This paper first describes a remotely delivered education program called remote consulting in primary health care (REaCH) aimed at equipping health care workers in LMICs with knowledge, skills, and confidence to conduct remote consulting. We then present a 2-phase approach to evaluation: (1) a pretest phase to establish technical and face validity, and (2) our feasibility study of the delivery of the REaCH training to registered health workers and its cascade to other health workers, and the perceived impact of training on the delivery of health care remotely.

### REaCH Training Program and Its Development

REaCH training aims to equip health workers with an understanding of the variety, benefits, challenges, and organizational changes associated with remote consulting and the skills for implementation of remote consulting in their health care facilities. The training was developed in partnership between St Francis University College of Health and Allied Sciences (SFUCHAS) (Tanzania), King’s College London (KCL, UK), and the University of Warwick (UK). The REaCH training, and a sample presentation of the training materials, can be freely accessed on a not-for profit basis at the Warwick Medical School website [19].

The REaCH training, developed in April and May 2020, is designed for registered health workers (eg, nurses, doctors, clinical medical officers) with access to smartphones, at least intermittent access to Wi-Fi, and an ability to learn in English. We refer to these trainees as tier 1 trainees. They engage in self-directed learning using written and video materials. Activities and assignments are included, which encourage trainees to apply what they learn to their local context. Training materials are in English and can be downloaded as PDF files where network access is challenging. A facilitator introduces the 8-module course to the trainees and interacts with them via a social media platform to discuss the learning and assignments. Each module is designed to take 1-3 hours. The facilitator supports these tier 1 trainees to cascade their learning to health workers in their local team (tier 2 trainees) using the train-the-trainer approach. It is left to the discretion of the tier 1 trainees to decide what learning to cascade to the tier 2 trainees. Tier 2 trainees need to own a feature phone (ie, no internet or up to 2G enabled). In our pilot, the learning cascade was completed in the local language, Swahili.

The content of each module is described in Table 1. REaCH is delivered via Moodle [20], an open-source blended-learning app. For the facilitated discussions, in our pilot, we used WhatsApp (Meta Platforms) [21] as it was popular locally and content is encrypted; trainees did not share patient information on the group. An information and communication technology (ICT) officer provided telephone support to trainees when they encountered difficulty with Moodle and suggested solutions when internet access was difficult (eg, travelling to a local village to download the materials).

We used the talent, resource, alignment, implementation, and nurturing (TRAIN) framework to optimize our train-the-trainer approach [22]. The facilitators who delivered the tier 1 training and the tier 1 trainees themselves were health professionals willing and able to train others (*talent*). We provided airtime and internet for facilitators, and each tier 1 trainee received £60 (US $74.30) for airtime and internet (*resource*). We provided tier 1 trainees with a certificate of course completion so they could add this to their training portfolio (*alignment*). Embedded within the REaCH training are teaching and activities related to implementation of remote consulting and how to cascade learning (*implementation*). There is opportunity for the tier 1 trainees to maintain contact on social media after the course for peer support (*nurturing*).

The facilitator is supported by a facilitator’s guide incorporating pedagogical principles underpinning the course, logistics, expectations, and tips to optimize trainee engagement. The learner is provided with a guide covering learning expectations, how to seek help, how to organize cascade training, and other logistical issues.

In July 2020, we pretested the first iteration of the REaCH Moodle course to establish technical and face validity with university-based professionals, 11 from SFUCHAS and 1 from the United Kingdom. The test demonstrated that it was possible and acceptable to use Moodle for delivering the course.

Based on feedback from this test, we included the WhatsApp group for facilitator support, developed the facilitator and trainee guides, and notes on how to cascade each module, an introductory video, and the option of downloading course materials as PDF files to enable studying to continue when digital access was interrupted. This version of REaCH was used in the second iteration feasibility study (August 2020) described in this paper. During this period, we obtained funding to support the airtime requirements of leaners to undertake and cascade training and deliver remote consultations to their patients. This timeline is presented in Table 2.

**Table 1:** REaCH^a^ modules.

Module	Description
Introduction	Why is remote consulting important?
1	What devices and platforms are used in remote consulting?
2	How does my role change and the care I provide my patients?
3	What are the risks and benefits of remote consulting?
4	What patient outcomes can I expect, including limiting COVID-19 spread?
5	What new issues arise in remote consulting that are different from face-to-face care?
6	What is my plan for delivering my work remotely (and that of my team/colleagues)?
7	How can I evaluate my own remotely delivered health care practice (and that of my team/colleagues)?
8	How can I influence others to change to remote consulting?

^a^REaCH: remote consulting in primary health care.

**Table 2:** Timeline: REaCH^a^ development, training process, and feasibility study.

Period	Training phase	Details
April-May 2020	Training development	REaCH training developed for online delivery (using Moodle and mobile devices)
July 2020	Pretesting	REaCH training pretested with university-based professionals in Tanzania (n=11) and the United Kingdom (n=1) Online training delivery mode found to be acceptable
July-August 2020	Adaptation	REaCH training adapted to include: Use of WhatsApp Facilitator and trainee guides Cascade notes Introductory video Downloadable materials
August-September 2020^b^	Feasibility study (as reported in this paper)	REaCH training delivered to 12 tier 1 trainees, Ulanga District Training completed by 12 trainees Evaluation completed by 10 (83%) trainees
		Cascade training delivered by 9 (75%) trained tier 1 trainers to 63 tier 2 trainees
August 2020-March 2022	Trial	Stepped-wedge trial of REaCH training in Tanzania and Nigeria underway

^a^REaCH: remote consulting in primary health care.

^b^Data collected and analyzed in this paper.

### Feasibility Study Objectives

The feasibility study objectives were to evaluate the trainees’:

- Response to REaCH training, their engagement levels, and their perceptions of the content and process (reaction)
- Perceptions of their level of understanding of the topic, including knowledge, skills, and attitudes, after undertaking the training (learning)
- Intended changes in how they deliver health care after completing the training and how the training contributed to this (behavior)

## Methods

### Study Design

**Figure 1:**
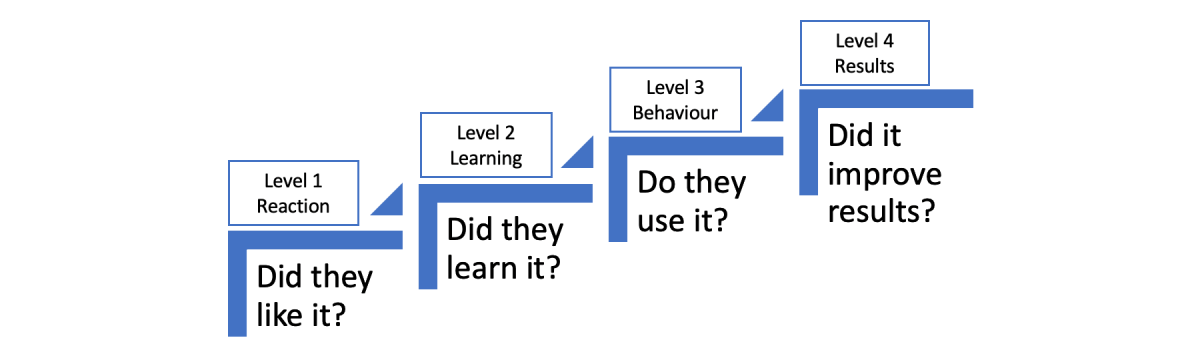
Summary of 4 levels of Kirkpatrick’s model.

In this feasibility study, we implemented and then evaluated the REaCH training using a survey, qualitative interviews, and documentary analysis. Our study was informed by Kirkpatrick’s model [23] for assessing informal and formal learning (Figure 1).

We assessed the *reaction* and self-reported *learning* and intended *behavior* change.

### Ethical Considerations

We used the Frascati definition of research, as summarized by the University of Warwick, to determine whether this study was considered research. We considered it not to be research, as its purpose was testing and standardization [24].

We subsequently checked our decision using the UK Medical Research Council and National Health Service (NHS) Health Research Authority tool for assessing ethics review, which indicated we did not need ethical review [25].

We received permission from the district medical officer (Ulanga District) for the participants (health care workers) to participate in the training and its evaluation.

### Trainees and Setting

Tier 1 trainees were enrolled from health facilities in Ulanga District of remote rural Tanzania. Ulanga has a population of 265,203, with 1 hospital, 2 health centers, and 23 dispensaries [26,27]. Tier 1 trainees were selected using purposive and referral sampling and fulfilled the following criteria: they consulted with patients, worked in a rural area, owned a smartphone or computer, had access to Wi-Fi, were prepared to include remote consultations by phone as part of their health care practice, and were willing to cascade training to 7 other health workers in their team.

Tier 2 health workers were enrolled by the tier 1 trainees. They had to consult with patients, own a feature phone, and be prepared to add remote consultations by phone to their health care practice. The training was delivered between August 10 and September 2, 2020. All trainees received information about the evaluation and verbally consented to it.

### Data Collection

#### The Survey

Questionnaires were developed by authors TM and SP for completion by trainees after each module and at the end of the training. These were structured around Kirkpatrick’s model of learning [25], with Kirkpatrick’s second and third levels (learning and behavior) assessed by self-report. We asked trainees about the process of undertaking the training (dichotomous questions and open-ended questions) and about their satisfaction (reaction), learning, and any intended changes to health care delivery as a result of the training (behavior) using 5-point Likert scales. A link to the survey was emailed to trainees and completed via SurveyMonkey [28].

#### Qualitative Interviews

The facilitator, all tier 1 trainees who completed the training, and, from each of their groups of tier 2 trainees, a convenience sample of 2 tier 2 trainees were invited for in-depth semistructured interviews. These were conducted by telephone following completion of the training by a researcher (TM) experienced in qualitative methods. Interviews explored participants’ perceptions and experiences of the training and their views about remote consulting. Each interview was recorded digitally, transcribed verbatim, and translated by this researcher.

#### Documentary Analysis

We collated WhatsApp texts and emails between facilitator and trainees and reports written by tier 1 trainees after they had cascaded the training to tier 2 trainees. The tier 1 trainees reported on their experiences of cascading training, including topic selection, duration of training, preparedness for teaching and learning, how they motivated the tier 2 trainees, and advantages and disadvantages of the REaCH Moodle training approach.

### Data Analysis and Trustworthiness

The survey results were analyzed descriptively. Interview transcripts were independently coded and analyzed thematically [29] by 3 team members (authors TM, BC, AD). Coding disagreements were resolved through discussion within the wider research team. TM analyzed the written documents thematically [29]. The research team held weekly debriefing meetings to reflect on the training and evaluation, identify/respond to challenges, share insights, and collectively make sense of the data [30]. We triangulated and integrated our findings in interpretation [31].

## Results

### Trainees

In total, 12 tier 1 trainees were enrolled within the REaCH training program, 3 (25%) women and 9 (75%) men. Tier 1 trainees were predominantly senior medical figures in participating health facilities (mostly doctors or assistant medical officers). In addition, 63 tier 2 trainees received cascaded training. The tier 2 trainees included a variety of health practitioners in the region: clinical officers, nurses, medical attendants, community health workers, and pharmacists, as well as 3 (5%) laboratory technicians and 2 (3%) radiologists, who were anecdotally delivering remote consulting. Of the 63 tier 2 trainees, 24 (38%) were women. Trainee characteristics are presented in Table 3. Of the 12 tier 1 trainees, all completed the training; however, 2 (17%) faced delays due to difficulty with internet connection and subsequently did not complete the evaluations or the cascading process. In addition, 1 (8%) tier 1 trainee faced personal circumstances, which precluded them from completing the cascading process. Thus, 9 (75%) tier 1 trainees cascaded their training to tier 2 health workers in their teams (N=63).

**Table 3:** Trainee characteristics.

Cadres	Tier 1 trainees				Tier 2 trainees	
	Enrolled (N=12), n (%)	Training completed (N=12), n (%)	Training cascaded (N=9), n (%)	Enrolled (N=63), n (%)		Training completed (N=61), n (%)
Medical doctors	6 (50)	6 (50)	5 (56)	1 (2)		1 (2)
Assistant medical officers	5 (42)	5 (42)	3 (33)	2 (3)		2 (3)
Clinical officers and assistant clinical officers	N/A^a^	N/A	N/A	18 (29)		18 (30)
Pharmacists	1 (8)	1 (8)	1 (11)	3 (5)		3 (5)
Community health workers	N/A	N/A	N/A	2 (3)		2 (3)
Radiologists	N/A	N/A	N/A	2 (3)		2 (3)
Laboratory technicians	N/A	N/A	N/A	3 (5)		3 (5)
Nurses	N/A	N/A	N/A	27 (43)		25 (41)
Medical attendants	N/A	N/A	N/A	5 (8)		5 (8)
Gender (female)	3 (25)	3 (25)	3 (33)	24 (38)		23 (38)

^a^N/A: not applicable.

### The Survey: Training Process Questionnaires

Survey questions about the process of training were completed by 9 (75%) of 12 tier 1 trainees; 3 (25%) trainees were unable to complete the survey due to a poor internet connection.

All 9 responding tier 1 trainees had studied in their own personal time; 7 (78%) said that they also studied during working hours, 8 (89%) had completed all 8 REaCH modules, and 1 (11%) respondent completed 5 modules. All completed the assignments associated with the modules studied. Respondents spent 1-3 hours studying per module. Of the 9 respondents, 5 (56%) completed these modules in the allocated 6-day time frame, while the other 4 (44%) completed it within 8 days. Delays were due to busyness, device and network challenges, and initial low technological competence. All respondents found the assistance from the ICT officer and facilitators to be effective and timely.

### The Survey: Reaction, Learning, and Behavior Questionnaires

Survey questions on reaction, learning, and behavior [23] were completed by 10 (83%) of 12 respondents (see Multimedia Appendix 1). There was little disagreement with the questions. All 10 respondents agreed that the training was useful and that facilitation was sufficient and timely. All 10 respondents appreciated the online and WhatsApp method of teaching and found that the learning outcomes were realistic and achievable. In addition, 9 (90%) of 10 respondents would strongly recommend this type of training to other health care workers.

Every respondent reported receiving the skills needed to learn remote consulting and to apply these skills to their jobs. They each reported already using the training in their daily work and being able to train other health care workers in this content.

### Qualitative Interviews

Telephonic interviews were carried out with the tier 1 training facilitator, 9 (75%) tier 1 trainees, and 16 (25%) tier 2 trainees. Interviews lasted between 15 and 30 minutes each, with tier 1 interviews conducted in English and tier 2 interviews in Swahili. See Multimedia Appendix 2 for the interview question scaffold.

We present the results under the following themes: perceptions of the REaCH program, challenges encountered during the training, learning from REaCH training, how the training could be improved, and trainees’ views on implementation of remote consulting into their routine practice. Trainees are labeled according to their tier of training and the order in which they were interviewed, as follows:

- Tier 1: participant A, B, C,…
- Tier 2: participant AA, BB, CC,….

### Perceptions of the REaCH Training Program

Overall, the trainees appreciated the program and recommended continuation and expansion among their peers.

I wish to congratulate the initiators of this program. I would recommend this knowledge to be taught in the health colleges so that we now begin to recruit new doctors with high experience in remote consultation.

Tier 1, participant D

Generally, the participants perceived the course as a good course, something [that] is also a success.

Tier 1, facilitator

### Challenges Encountered During the Training

Over half of the tier 1 trainees reported challenges with their digital technology, including storage capacity of smartphones, low technological competence, and network challenges.

There is no stable internet connection in this area, and this was 1 of the challenges I faced during the training. Ooh, likewise, the mobile network we use in our area is not stable.

Tier 1, participant E

Nevertheless, trainees found the assistance from ICT personnel and facilitators to be effective and timely.

There were several technical challenges and some issues concerning the arrangements of the modules; therefore, we used to seek instruction from the facilitator and ICT personnel, [and] actually, they were responding as soon as possible. We were told how to download the modules and the way we could go about reading them.

Tier 1, participant A

Although some trainees found it difficult to schedule the training around their work, others appreciated the flexibility of the online training.

The shortage of enough time…I spent many hours at work, so I had to make sure I read the modules in my extra time.

Tier 1, participant D

The training time planning was well arranged because it allowed us to engage in learning at any daily time.

Tier 2, participant CC

### Learning From REaCH Training

All participating health care workers felt that their knowledge increased and that their behavior had changed since the training program. Some trainees were learning about remote consultation for the first time.

Yes, there are some changes; as you know, the modules have insisted on practicing remote consultation instead of face-to-face consultation, which we only trusted before. Recently, we have noticed that remote consultations are also appropriate, and actually, this alternative will work properly…!

Tier 1, participant E

Many remarked upon the usefulness of remote consulting during COVID-19.

Actually, this would assist much during this time of COVID-19 spread because it avoids the chance for having physical interaction between the patients and doctors.

Tier 1, participant F

Over half of the interviewees reported paying attention to privacy and confidentiality during remote consultations.

I do the remote consultation in a professional way by making sure I ask for consent, ensuring privacy as well as keeping their records, and making sure I continue to make follow-up on the patient's progress.

Tier 1, participant A

Trainees reported talking to patients before they attended clinic and following them up by phone rather than face-to-face. This included conducting remote consultations for patients who hesitated to attend face-to-face consultations out of fear of stigmatization.

I have started to offer advice to patients with shameful diseases remotely. You know the patients with gonorrhea can feel free to talk to a health care provider remotely rather than face-to-face.

Tier 1, participant H

Trainees had worked out how to bill for remote consultations.

I enjoyed this learning style because I have discovered that through this…this can give us an extra alternative to get money!

Tier 1, participant D

### Recommendations for Improvement of the REaCH Training

Tier 1 trainees recommended a face-to-face meeting at the beginning of the course and additional time at the start to familiarize themselves with Moodle.

My advice is this…we should be making face-to-face meetings at least once at the beginning of a course that will be helpful in making things more clear! We can be taught physically on how to go through the Moodle and students’ forum as well.

Tier 1, participant D

The facilitator agreed that additional time was needed at the start of the course for familiarization and supported the tier 1 trainees in including this during cascading. The facilitator supported some tier 1 trainees in producing printed materials for the tier 2 trainees. However, we found that many of the tier 2 trainees had smartphones and received the online materials easily.

The first thing that has been successful in this training [is] the majority of us received the learning materials on time simply because we have got smartphones through which we received them.

Tier 2, participant MM

The facilitator was keen to see the addition of incentives to engagement, such as accreditation and payment for time spent undertaking the training.

### Implementation of Remote Consulting in Routine Practice

To further apply their learning in practice, trainees said they needed airtime and internet packages, suitable electronic devices, and improved infrastructure.

Our digital devices are not modern ones...we should be assisted with the internet packages to support online processes during the moment of interacting with the remote clients.

Tier 1, participant B

We can’t provide the remote consultation if the supporting infrastructures like mobile networks are not working very well. Therefore, the government should ensure all necessary infrastructures for remote consultations.

Tier 1, participant I

Trainees emphasized the importance of governmental recognition to ensure adequate compensation for their work.

This needs some money…workers should be paid for this extra duty.

Tier 1, participant D

They recognized the need to inform the community about remote consulting.

Moreover, we need to make the community be aware and recognize this kind of consultation.

Tier 1, participant I

The lack of pharmacies and pathology laboratories in rural areas was identified as a barrier to successful remote consulting.

First of all, it will be difficult to make a physical examination, and the second challenge will be a shortage of pharmacies in remote areas, something [that] will make the remote clients fail to get medicines after consultation.

Tier 2, participant CC

The government should allow individuals to establish laboratories in remote areas. You know there are many laboratories that have been stopped due to the fact that they don’t meet the eligibility requirements. So, we should have enough laboratories in rural areas so that clients may have test[s].

Tier 2, participant DD

Trainees noted that some members of the community would not have easy access to a phone, as they are owned by the heads of families and sharing phones can reduce confidentiality.

You know most of the mobile owners in the family level are heads of the families; therefore, the other family members will not be free enough to use those phones. So far, sharing phones will reduce the confidentiality of the clients’ information.

Tier 2, participant MM

Older members of the community were unlikely to afford a phone, and there were community members who were illiterate and so unable to use text messaging.

Most of the community members, especially elders, are not possessing mobile phones, so they can’t make consultations by themselves without asking the help from their neighbors.

Tier 1, participant J

Generally, this is a good idea, but I am doubting whether the elders will afford to pay the costs for remote consulting.

Tier 2, participant NN

So far, some of them are not aware of reading and writing; therefore, they can’t send a text to the doctor when required to do so.

Tier 1, participant J

Some trainees were concerned with how to keep records of remote consultations.

We are still lacking the best alternative to keep the remote clients’ records…we should find how to solve this challenge.

Tier 1, participant B

One said there needed to be a different way of referring patients between health care workers when they were consulting remotely.

To make referrals to remote clients, there should be an alternative for facilitation referrals from junior to senior HCWs [health care workers].

Tier 1, participant L

Qualitative findings from the interviews with tier 1 and 2 trainees were compared with analysis of the reports on the cascading process by tier 1 trainees.

### Documentary Analysis: Tier 2 Training Process

Tier 2 training was completed over 3 days, with 2 pretraining preparation days where tier 1 trainees informed the tier 2 trainees about the aims of the course, its contents, and the training style and answered any questions. Tier 1 trainees selected modules 1, 2, 3, 5, and 7 for cascade as they were deemed to be the most clinically relevant (Table 1).

Introduction to training occurred via phone conferences and WhatsApp chats, and learning was primarily conducted through smartphone and featured phones with phone calls, texts, phone conferences, WhatsApp message group, and emails.

Soon after receiving the modules, the [tier 2] trainees started learning independently…when issues could not be understood, they used to make calls and send texts for more discussion and elaboration.

Tier 1, participant E

Where there were unstable internet connections, tier 2 trainees traveled to their nearest colleagues to pick up the module PDF files or to a nearby area with a stronger internet connection.

The participants from the areas with [an] unstable internet connection were advised to move to the areas with [an] internet connection in order to download the materials.

Tier 1, participant A

Of the 63 tier 2 trainees enrolled, 61 (97%) trainees completed the course, with 53 (87%) completing it within 3 days, while 2 (3%) did not complete the course due to personal reasons.

One participant’s child got sick during the week of training that made her fail to complete the training in time.

Tier 1, participant F

Modifications used to ensure engagement included using reminder texts and phone calls to gauge and maintain attention of the trainees, using group discussions to increase teamwork, and conducting face-to-face conversations when the trainees and trainers were working in the same health facilities.

Sometimes, we were sending texts through the phone and WhatsApp media to remind them about the discussion time.

Tier 1, participant D

I also used to put some question in [the] WhatsApp platform to assess the trainees’ understanding.

Tier 1, participant F

Participants’ chats in WhatsApp assisted to assess the participation rate. You know, we were making calls to [the] training facilitator once per day to report on cascading progress and share the technical experience.

Tier 1, participant B

Modifications to solve logistical issues included translating the training documents into Swahili to overcome language barriers, providing downloadable materials that trainees could access from nearby villages when they had unstable internet connections, and moving group calls to early morning and evening hours to avoid working hours.

All in all, when I posted learning materials on the WhatsApp media, I tried to elaborate in Swahili to make them understand the contents.

Tier 1, participant A

## Discussion

### Principal Findings

This feasibility study found that remotely delivered professional REaCH training [19] using the Moodle app supported by cascade training infrastructures is technically and pedagogically feasible and well received by trainees in rural Tanzania. They were satisfied with the course and would recommend the program to other health care workers (reaction). They expressed that they learned skills needed to remotely consult within the health system, including how to bill patients for the consultations, and they were able to cascade the teaching (learning). Trainees reported confidently implementing remote consulting and increased understanding of topics, such as medical ethics of remote consulting and behavior change theory (behavior) [23].

Barriers to remote consulting implementation identified by our trainees include lacking digital infrastructure and few resources, inflexible billing and record-keeping systems, and limited community awareness about remote consulting. Having local technical support for learners proved invaluable to delivery and receipt of training. The greatest immediate barrier to supporting both the upscaling of REaCH training in LMICs and subsequently the delivery of safe and trustworthy remote health care is the cost of the data or airtime for the health workers themselves.

### Comparison to Prior Work

Our REaCH training responds to a need identified by current research. In a systematic review of 14 studies assessing the feasibility and efficacy of remote consulting in LMICs, all studies identified that with adequate training, health care workers were able to learn to use mobile phones to deliver health care, but the review emphasized that sufficient initial and ongoing training is required to support the implementation of remote consulting [32]. In a systematic review of the barriers to remote consulting, lack of training was likewise identified as a key barrier [33]. Furthermore, during the COVID-19 pandemic, in a survey of physicians in Libya, 638 (94.8%) of 673 of participants expressed willingness to participate in a telemedicine training course [34].

Ediripulge et al. [35] literature review of 9 studies that described the delivery of training in telehealth not only emphasized the importance of adequate training to ensure integration of remote consulting in health systems but also found that the programs were predominantly conducted online and were a mixture of continuous professional development and university courses [35].

A scoping review, published after the development of the REaCH curriculum, describes the range of topics covered by courses that train health personnel for remote consulting [36]. Our course covered the key topics commonly taught, and included topics less commonly taught, including ethics, professionalism, and challenges of remote consulting. In this review, only 2 (5%) of 43 studies were conducted in LMICs [37,38]. One of these papers, similar to our study, evaluated its program using Kirkpatrick level 3 evaluation, while the other paper also included Kirkpatrick level 4 evaluation [39,40].

As in our study, the train-the-trainer approach to remote consulting education was successfully used in Rwanda to train community health care workers in monitoring pregnancy and pregnancy-related complications remotely and in Liberia to upskill traditional midwives to use mobile technology for short messaging service (SMS) texting [39,40]. Also replicated in our findings, remote delivery of remote consulting training in Brazil and India has been successful [38,41]. These trials and other similar remote consulting training programs in LMICs have been well received with high completion rates, as with our pilot study [42,43].

### Strengths and Limitations

This study has some key strengths. We tested the program at several stages, undertook intensive evaluation at each stage, and were thus able to improve the program multiple times. We collected quantitative data and qualitative data to evaluate the training.

REaCH training and its pilot evaluation were undertaken at speed in response to the urgent need to support Africa’s low-resource health care system in the face of the COVID-19 pandemic. Consequently, it has some limitations. The results are based on a relatively small number of health workers. Kirkpatrick’s model informed the evaluation, but the second and third levels (learning and behavior) were assessed by self-report, with no external observation or validation. Although Kirkpatrick’s model has its limitations for assessing medical education, it is useful for an evaluation, such as this that assesses immediate effects [44]. The survey questions were developed and delivered in a short time frame. Although each question captures 1 area of interest, some include 2 issues, which we are unable to tease out. There was a marked positive skew in the survey results, although in the exploratory semistructured interviews, respondents talked about both positives and negatives. The evaluation was conducted at 1 site, a single region in a single country, and by the team that developed the training.

We are currently running a stepped-wedge trial of REaCH training in Tanzania and Nigeria to evaluate actual *behavior* change and *results* in terms of the impact on health care delivery [45].

### Conclusion

The REaCH program, providing training on remote consulting, is feasible and acceptable and successfully initiated behavior change in health care workers in a rural district in Tanzania. Trainees identified a need for resourcing of data/airtime and a technical and device infrastructure for the implementation of remote consulting.

## Appendix

Multimedia Appendix 1 Survey responses aligned with Kirkpatrick’s model (n=10 respondents).

Multimedia Appendix 2 Interview questions scaffold.

## Abbreviations

ICT: information and communication technology
LMIC: low- and middle-income country
REaCH: remote consulting in primary health care
SFUCHAS: St Francis University College of Health and Allied Sciences
