# Supplementary material for: Remote Consulting in Primary Health Care in Low- and Middle-Income Countries: Feasibility Study of an Online Training Program to Support Care Delivery During the COVID-19 Pandemic
Source: JMIR Form Res. 2022 Jun 14;6(6):e32964. doi: 10.2196/32964 (PMC9200055; doi:10.2196/32964)
Supplement: Multimedia Appendix 1 [file formative_v6i6e32964_app1.docx]

Survey Questions

Survey responses aligned with Kirkpatrick’s model (n=10 respondents).

| **Questionnaire item** | **Strongly agree** | **Agree** | **Neither agree/disagree** | **Disagree** | **Strongly disagree** |
| --- | --- | --- | --- | --- | --- |
| *Satisfaction with REaCH (Reaction)* | | | | | |
| Was the learning environment for this self-directed online training conducive for learning? | 4 | 5 |  | 1 |  |
| Did you like the method and style (pace, online, WhatsApp etc) used to deliver this ‘REaCH’ course? | 5 | 5 |  |  |  |
| Was the presentation of the modules engaging and allowing the interaction with other participants? | 5 | 5 |  |  |  |
| Did you feel the training was useful and worth your time? | 6 | 4 |  |  |  |
| Were you receiving enough and timely assistance from the course facilitator and information and communications technology personnel during the training? | 5 | 5 |  |  |  |
| Was it easy to complete the modules, doing activities and assignments? | 7 | 2 | 1 |  |  |
| Were the learning outcomes realistic and achievable? | 7 | 3 |  |  |  |
| *How REaCH facilitated knowledge & skills (Learning)* | | | | | |
| Do you think you have received skills you needed to learn and you can apply them to your job? | 5 | 5 |  |  |  |
| Did your work environment contribute to your ability to learn? | 5 | 5 |  |  |  |
| Would you recommend this type of training to other health care workers? | 9 | 1 |  |  |  |
|  | **Yes** | **No** | **Free text** | | |
| Are they modules you would like to pursue learning further? | 7 | 3 | Differences between remote and f2f care; Benefits to patient outcomes; planning for remote care delivery; COM-B model and resistance to change [1]. | | |
| Were there any other internal or external obstacles which affected your training? | 6 | 4 | Device and internet network; work interruptions; Moodle technical issues | | |
| Did anything noticeable promote your ability to learn during the training? | 9 | 1 | Facilitator support; funds for data/airtime; topic importance; peer learning. | | |
| *Perceived behaviour change* | | | | | |
| **Item** | **Strongly agree** | **Agree** | **Neither agree/disagree** | **Disagree** | **Strongly disagree** |
| Can you confidently cascade (train) what you learned to the other health care workers? | 6 | 4 |  |  |  |
| Are you using what you learned in training in your daily work? | 5 | 5 |  |  |  |

Reference

1. Michie S, van Stralen MM, West R. The behaviour change wheel: a new method for characterising and designing behaviour change interventions. Implement Sci. 2011;6:42-. PMID: 21513547. doi: 10.1186/1748-5908-6-42.

This is a Multimedia Appendix to a full manuscript published in the J Med Internet Res. For full copyright and citation information see <http://dx.doi.org/10.2196/jmir.32964>
