# Supplementary material for: Remote Consulting in Primary Health Care in Low- and Middle-Income Countries: Feasibility Study of an Online Training Program to Support Care Delivery During the COVID-19 Pandemic
Source: JMIR Form Res. 2022 Jun 14;6(6):e32964. doi: 10.2196/32964 (PMC9200055; doi:10.2196/32964)
Supplement: Multimedia Appendix 2 [file formative_v6i6e32964_app2.docx]

Interview questions scaffold.

**Interview Guide to Tier 1**:

**Interviewer:** Doctor, as I told you, we are going to have a simple discussion concerning ReaCH training. I understand that you are among the trainees who have attended this course, therefore I wish to know your experience concerning this training and so on, welcome!

Process evaluation

I: Please tell me, how did you get engaged in this course?

I: Can you please explain your experience in using Moodle and WhatsApp in this training?

I: Tell us about any changes which required you to use more learning alternatives apart from the planned ones in this training (Moodle and WhatsApp)?

I: Did you make phone calls to your facilitator while learning?

I: How did you interact with your trainer during the learning process?

I: How did you complete your modules and other course-related activities?

I: What are the challenges you may have faced in completing this course?

Reaction and Learning evaluation

I: How was your completion rate, and time spent on this self-directed online training course?

I: In which way did the training address the needs in your role as a health care worker who makes consultations?

I: Did the training style work for you? Consider pace, delivery method, location (in-person or online), content, etc. (how?)

Behaviour evaluation

I: In order to apply what you have learned, what resources or support do you need?

I: Are there noticeable changes in individual and team performance post-training?. (if, yes) Please explain those changes!

I: Think back to prior training. How are you performing in your role now compared to the previous one?

I: Are there any obstacles or challenges that would prevent you from using your new skills efficiently? ***(if yes)*** What are they?

I: What can be done to make you feel motivated to use the new skills you’ve learned?

I: May you give us your general comments, views or suggestions on this course?

I: Thank you and this is the end of our discussion

**Tier 2 Interviews**

I: Hello, my name is ……., I am one of the research team members from St. Francis University College for health and allied sciences. I understand that you are among the REaCH course trainees therefore I wish to hear your views concerning this training.

I: Please tell me, what were the biggest strengths and weaknesses of this training?

I: What are three important things you learned from this training?

I: In which way did the training address your role as a health care worker who makes consultations?

I: In order to apply what you learned, what resources or support do you need?

I: Are there any obstacles or challenges that would prevent you from using your new skills efficiently?

I: What can be done to make you feel motivated to use new skills you have learned?

This is a Multimedia Appendix to a full manuscript published in the J Med Internet Res. For full copyright and citation information see http://dx.doi.org/10.2196/jmir.32964
